# Tablet Disintegratability: Sensitivity of Superdisintegrants to Temperature and Compaction Pressure

**DOI:** 10.3390/pharmaceutics14122725

**Published:** 2022-12-06

**Authors:** Audrey Yi Zheng, Paul Wan Sia Heng, Lai Wah Chan

**Affiliations:** GEA-NUS Pharmaceutical Processing Research Laboratory, Department of Pharmacy, National University of Singapore, 18 Science Drive 4, Singapore 117543, Singapore

**Keywords:** disintegration, temperature, superdisintegrant, swelling, strain recovery, tablet

## Abstract

Tablet disintegration is an important pre-requisite for drug dissolution and absorption. The disintegration test is typically conducted at 37 °C, but the intragastric temperature may vary due to meals or fever. This study investigated the effects of temperature and compaction pressure on tablet disintegratability to gain deeper insights into superdisintegrant sensitivity and function. Tablets with either sodium starch glycolate or crospovidone as disintegrant were prepared at various compaction pressures and subjected to the disintegration test using different medium temperatures. Preheating of tablets was also employed to establish instant temperature equilibrium between the tablet and the disintegration medium. Liquid penetration and disintegration were faster as the medium temperature increased or compaction pressure decreased. Swelling or strain recovery disintegrants exhibited similar sensitivity to variations in the medium temperature. Preheating of the tablets resulted in slower disintegration, but this effect was reversible upon cooling, hence the slower disintegration was unlikely to be attributed to changes in the disintegrant physical state. The temperature difference between the tablet and the disintegration medium likely affected the rate of fluid flow into tablets and influenced disintegration. Understanding disintegrant temperature sensitivity would help to avoid unacceptable fluctuations in disintegration due to temperature variations. The temperature difference effect could also be harnessed to boost disintegrant performance.

## 1. Introduction

Tablets are the most common and preferred dosage form for administering medications to patients [1,2]. Tablet disintegration refers to the breakup of a compressed tablet into multiple particles when it comes into contact with an aqueous medium [3]. Disintegrants are often included in tablet formulations to promote the breakup of tablets in order to to increase the surface area available for drug dissolution [2]. Earlier known disintegrants are plant-derived polymeric materials such as starch and microcrystalline cellulose, but their presence in tablets was often fortuitous as it was not a requirement for tablets to show disintegratability. With the later appreciation of biopharmaceutical properties of dosage forms, tablet disintegration became mandated. Over time, more efficient disintegrants were developed and marketed. Of particular commercial success are the very efficient disintegrants introduced, referred to as superdisintegrants. Examples of superdisintegrants include sodium starch glycolate (SSG) and crospovidone (XPVP).

Various mechanisms of disintegrant action have been proposed, and the more commonly mentioned are swelling, strain recovery and wicking. Swelling refers to the omnidirectional volume expansion of disintegrant particles upon contact with water [4,5]. Strain recovery is the reversible viscoelastic process of deformation [6]. The expansion due to strain recovery is unidirectional and in the opposite direction of the compaction force exerted [4]. SSG acts mainly by swelling, whereas XPVP acts mainly via strain recovery. Wicking refers to the process of liquid entry into the tablet by capillary action [2,7], providing water that is required for swelling, strain recovery and other disintegrant mechanisms [2,8,9,10]. Wicking can also cause weakening of the tablet structure by disrupting hydrogen bonds between particles [11]. Disintegrants and other hydrophilic components within the tablet help to confer hydrophilicity to the matrix and contribute to liquid penetration [12]. The main mechanism of disintegration differs from one disintegrant to another [13].

One of the main factors affecting tablet disintegration is the type and concentration of disintegrant used in the formulation [14]. The compaction pressure also has a significant impact on tablet disintegration. Generally, the greater the compaction pressure, the longer the disintegration time will be [15,16,17]. This is because higher compaction pressures form stronger bonds between particles, and these bonds will take a longer time to be disrupted during a disintegration test. A higher compaction pressure also reduces tablet porosity, which in turn impedes liquid penetration, thus delaying tablet disintegration [15,16,17]. However, when tablets are very porous, disintegrant efficacy may be diminished as the swelling pressure of disintegrant due to volumetric expansion is partially nullified by accommodation in the large void spaces present. In essence, when the compaction pressure is too high or too low, tablet disintegration could be prolonged. Interestingly, higher compaction pressures were reported to hasten disintegration of tablets containing XPVP [18]. This was attributed to its strain recovery mechanism, which is promoted at higher compaction pressures.

The USP disintegration test is conducted at 37 ± 2 °C to simulate the physiological temperature [19]. However, body temperature is variable and may rise to over 40 °C during febrile episodes [20]. Furthermore, tablets are usually recommended to be taken with a glass of water but normally, neither the volume nor temperature of the water is specified. Thus, some patients may choose to consume the tablet with water at room temperature while others, with a cold or hot drink. Moreover, certain medications are meant to be taken after meals and the meals consumed may alter the intragastric temperature. As a result, tablets that are ingested may often encounter temperatures outside of the compendial disintegration test temperature of 37 °C. In a study conducted by Sun et al. [21], it was found that the intragastric temperature reached 43.0 °C after consuming a hot drink (50 °C) and 21.2 °C after a cold drink (4 °C), and returned to baseline after 20 to 30 min. Almukainzi et al. also reported that tablet disintegration was slower in cold beverages and faster in hot coffee [22], demonstrating that the beverage or meal temperature could significantly influence tablet disintegratability.

Although the majority of the researchers observed that an increase in the disintegration medium temperature promoted tablet disintegration [23,24,25,26,27,28,29] due to the increased rate of swelling and liquid penetration [24,30], other researchers reported that the disintegration time was unaffected by the disintegration medium temperature [31,32]. The diversity in literature findings could be due to differences in the type of disintegrants and tableting conditions employed. Although much research has been conducted regarding the effect of the disintegration medium temperature, the relative sensitivity of disintegrants to variations in the disintegration medium temperature has not been investigated in great detail. In particular, superdisintegrants are increasingly used in tablet formulations due to their high disintegrant efficiency, and hence, it was of interest to gain deeper insights into their performance. This study aimed to investigate the effect of the disintegration medium temperature on the disintegratability of tablets containing superdisintegrants with different mechanisms of action. The interplay between the disintegration medium temperature and compaction pressure, as well as the influence of tablet temperature were also investigated.

## 2. Materials and Methods

### 2.1. Materials

Sodium starch glycolate (SSG; Primojel, DFE Pharma, Goch, Germany) and crospovidone (XPVP; Kollidon CL, BASF, Ludwigshafen, Germany) were the superdisintegrants employed in this study. Dicalcium phosphate dihydrate (DCP; Emcompress, JRS Pharma, Rosenberg, Germany) was used as the filler. DCP was chosen as it is a commonly used diluent that does not exhibit aqueous solubility or strong wicking, which could be influenced by the disintegration medium temperature and confound the investigation. Magnesium stearate (M-125, Productos Metalest, Zaragoza, Spain) was used as the lubricant.

### 2.2. Preparation of Tablets

Tablets were produced using a compaction simulator (STYL’One, MedelPharm, Beynost, France) using 14-mm flat-face punches and die (Natoli Engineering, Saint Charles, MO, USA). The tablet formulation comprised 2%, *w*/*w* SSG or XPVP, 97%, *w*/*w* DCP, and 1%, *w*/*w* magnesium stearate. Tablets weighing 1 g each were compacted at 65, 97 and 130 MPa, and all tablets were aged for at least 24 h prior to their use for characterization tests.

### 2.3. Characterization of Tablets

#### 2.3.1. Determination of Tensile Strength and Porosity

The weight, thickness, diameter and breaking force of five tablets were measured at least 24 h after compaction using a weighing balance (Quintix 35-1S, Sartorius, Göttingen, Germany), thickness gauge (547-300S, Mitutoyo Corporation, Kawasaki, Japan) and hardness tester (TBF 1000, Copley Scientific, Nottingham, UK), respectively. The tablet tensile strength and porosity were calculated using Equations (1) and (2), respectively.

(1) $$\mathrm{Tensile}\;\mathrm{strength}=\frac{2F}{\pi \times d\times h}$$

(2) $$\mathrm{Porosity}=(1-\frac{\rho_{tablet}}{\rho_{true,mix}})\;\times \;100\%$$

where *F*, *d* and *h* are the measured breaking force, tablet diameter and tablet thickness respectively, and ρ*_tablet_* and ρ*_true,mix_* refer to the tablet density and true density of the mixture, respectively. ρ*_tablet_* was obtained by dividing tablet weight by its volume and ρ*_true,mix_* obtained using a helium pycnometer (Penta-pycnometer, Quantachrome, Boynton, FL, USA).

#### 2.3.2. Disintegration Test

The disintegration times of five tablets for each formulation and compaction pressure were determined individually using the USP disintegration apparatus (DT2, Sotax, Basel, Switzerland), with deionized water as the disintegration medium. The water bath was adjusted to various medium temperatures from 33 to 43 °C to study the impact of variations in the disintegration medium temperature. Complete tablet disintegration was defined as the state in which no tablet residue with a palpably firm core remained on the screen [19]. Disks were not used, as the use of disks in the USP disintegration apparatus may contribute to greater mechanical damage to tablets and mask differences in the tablet disintegration time [33,34].

As the tablets might take some time to attain the temperature of the disintegration medium, another set of tablets was preheated in the oven for 30 min at the same temperature as the investigated medium temperature prior to the disintegration test. This was to establish a temperature equilibrium between the tablet and the disintegration medium instantaneously, allowing the effect of the disintegration medium temperature to be studied with less potential confounding factors.

The effect of preheating tablets was also further expanded to preheating tablets to different temperatures from 25 to 45 °C prior to disintegration at 37 °C. Additionally, to determine if the preheating effect was reversible, the temperature (25 or 45 °C) and duration (30 or 120 min) of the preheating process were varied in a full factorial design. Two sets of three tablets were preheated and for one set, the disintegration test was conducted immediately after preheating while for the other, allowed to cool at room temperature for 1 h before the test. The disintegration time of the tablets not subjected to any heat treatment was also determined as controls.

#### 2.3.3. Liquid Penetration Test

**Figure 1:**
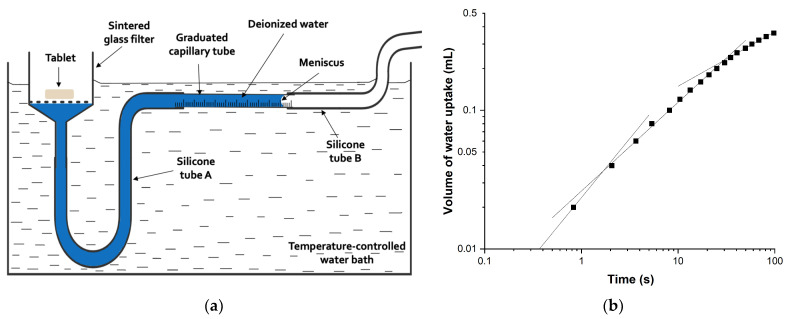
(**a**) Schematic diagram of the liquid penetration test apparatus, and (**b**) example of the double logarithmic water uptake plot with three linear portions.

Three tablets of each formulation produced at different compaction pressures were randomly selected for the liquid penetration test, using an apparatus consisting of a sintered glass filter (medium porosity) connected by a silicone tube A to a 2-mL graduated capillary tube and completely filled with deionized water from below the filter to the capillary tube (Figure 1a) [35]. The other end of the capillary tube was connected to the silicone tube B, and water was prevented from moving by fixing a clip at the open end. The capillary tube was positioned horizontally and on the same level as the base of the sintered glass filter, and a Whatman No. 1 filter paper placed on it. The set-up was immersed in a water bath controlled at 33, 37 or 41 °C, and the clip on the silicone tube B was removed.

The test was started by placing a tablet on the filter paper, and the time taken for the meniscus level to pass each graduation mark was measured using a stopwatch. The second linear portion of the double logarithmic water uptake plot (Figure 1b) was found to correspond to the filling of the capillary system within the tablet [35]. The slope of this portion of the water uptake plot was determined as the liquid penetration rate constant, which was used to compare the rates of liquid penetration into the tablet matrix for different tablets. The determination of the liquid penetration rate constant was conducted in triplicates and the results averaged.

#### 2.3.4. Moisture Analysis

The moisture content of a set of five tablets before and after preheating (to 25 °C or 45 °C for 30 min) was determined using a moisture analyzer (HE73, Mettler Toledo, Greifensee, Switzerland). The preheated tablets were immediately sealed in plastic bags upon removal from the oven to minimize any moisture reabsorption. The determination of moisture content for sets of five tablets was triplicated and results averaged.

### 2.4. Statistical Analysis

The results obtained were analyzed at 5% level of significance using a statistical software (SPSS Statistics 26, IBM, Armonk, NY, USA). The Kruskal–Wallis test was used to compare the disintegration time and liquid penetration rate of tablets at different medium temperatures, as well as that of tablets preheated to different temperatures. The Wilcoxon rank sum test was used to compare the disintegration time of tablets produced with SSG or XPVP as the disintegrant, as well as with or without preheating. Minitab 17 (Minitab Inc., State College, PA, USA) was used for the design of the experiment and generation of the contour plots.

## 3. Results and Discussion

### 3.1. Sensitivity of Superdisintegrants to Disintegration Medium Temperature and Compaction Pressure

**Figure 2:**
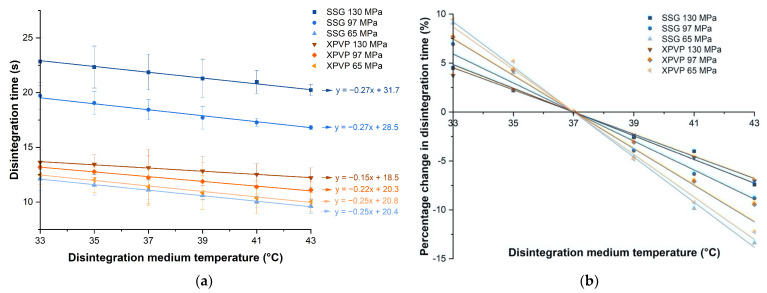
Plots of (**a**) disintegration time against disintegration medium temperature and (**b**) percentage change in disintegration time against disintegration medium temperature for tablets consisting of SSG and XPVP, produced at different compaction pressures. Note: All trend lines drawn are best fit lines.

**Figure 3:**
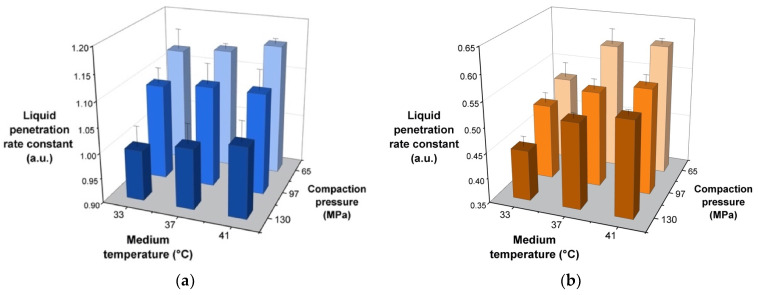
Plots of liquid penetration rate constant of tablets containing (**a**) SSG and (**b**) XPVP produced at different compaction pressures.

The disintegration time of tablets containing SSG or XPVP produced at different compaction pressures was plotted against the disintegration medium temperature (Figure 2a). As compaction pressure increased for either disintegrant, the disintegration time increased (*p* < 0.001). This could be attributed to the formation of stronger bonds between particles at higher compaction pressures, producing tablets with higher tensile strength (Table 1) that were more resistant to disintegration [15,16,17]. According to literature studies, the strain recovery mechanism of XPVP resulted in a more rapid disintegration at higher compaction pressures [18,36]. However, tablets consisting of XPVP in this study showed slower disintegration at higher compaction pressures, albeit the effect of compaction pressure prolonging disintegration was much less than for tablets with SSG. These conflicting results could be attributed to differences in the tablet formulations and tableting conditions. Further investigation showed that the liquid penetration rate of tablets containing XPVP in this study decreased with an increase in compaction pressure (Figure 3b). From the trend observed, it could be inferred that liquid penetration was a limiting factor for the disintegration of these tablets. The lower porosity (Table 1) hindered liquid penetration and outweighed the effect of strain recovery, hence tablets containing XPVP produced at higher compaction pressures exhibited slower disintegration.

The tablet disintegration time decreased as the medium temperature increased (Figure 2a). Although the tablets investigated in this study disintegrated rapidly, a consistent trend was observed for all tablets. The tablets exhibited an almost linear relationship between disintegration time and medium temperature (R^2^ ≥ 0.9) for all formulations compacted at different pressures. The effect of the medium temperature was statistically significant for both disintegrants (*p* < 0.05). The gradients of the best fit lines for tablets containing SSG or XPVP were approximately −0.3 and −0.2, respectively. This suggests that the sensitivity of SSG and XPVP to changes in the medium temperature was relatively similar, indicating that there is unlikely to be any significant difference in temperature sensitivity between the swelling and strain recovery mechanisms.

#### 3.1.1. Percentage Change in Disintegration Time Due to Medium Temperature

In order to better compare the effect of medium temperature on tablet disintegration time, the percentage change in the disintegration time was calculated with tablets disintegrated at 37 °C as the reference (Figure 2b). When the medium temperature decreased from 37 °C to 33 °C, the disintegration time increased by 5–10%. When the medium temperature increased from 37 °C to 43 °C, the disintegration time decreased by 7–15%. The percentage change in the disintegration time with the medium temperature was linear, with gradients ranging from −2.3 to −1.1% per degree temperature change (Figure 2b). This could be expected as the specific heat capacity of water has a constant value (~4.2 J/g °C) and an increase or decrease in temperature would mean a corresponding change in energy [37]. This meant that there was a constant quantum of energy per degree change for reaction (i.e., breaking of inter-particle bonds for disintegration). It can hence be inferred that the disintegration process is energetically dependent. It could be envisaged that tablet disintegration requires the disruption of a finite number of bonds, and the increased energy available for disruption would correspond linearly with temperature rise. Hence, there is an inverse relationship between the disintegration medium temperature and disintegration time.

#### 3.1.2. Liquid Penetration Rate into Tablets

It was postulated that the disintegration medium temperature can affect tablet disintegration through its effect on the rate of liquid penetration, in addition to bond disruption [24]. This was observed from liquid penetration tests which revealed that the liquid penetration rate into tablets generally increased as the disintegration medium temperature increased (Figure 3). In particular, tablets containing XPVP exhibited a greater increase in the liquid penetration rate as the medium temperature increased (*p* = 0.001). In contrast, the difference was less pronounced for tablets containing SSG and not found to be statistically significant (*p* = 0.644). When compaction pressure increased, the liquid penetration rate decreased, and this can be attributed to reduced tablet porosity. This was found to be significant for both disintegrants (*p* < 0.001 for SSG and *p* = 0.015 for XPVP). It was noted that in relative terms, the liquid penetration rate for tablets with XPVP was more sensitive to the medium temperature, while tablets with SSG were more sensitive to differences in compaction pressure.

#### 3.1.3. Combined Effects of Disintegration Medium Temperature and Compaction Pressure

**Figure 4:**
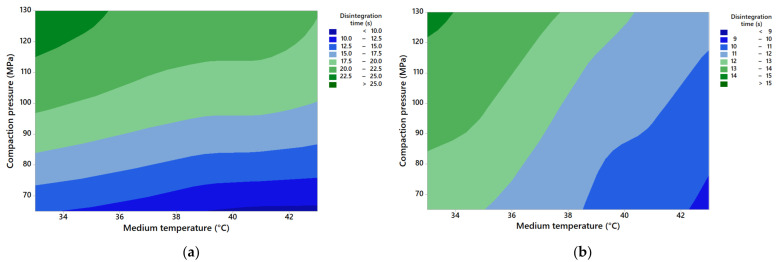
Contour plots of disintegration time against disintegration medium temperature and compaction pressure for tablets containing (**a**) SSG or (**b**) XPVP.

As tablets may be produced with a wide range of disintegrants, compaction pressures and other ingredients in the formulations should be carefully considered to avoid unacceptable fluctuation in tablet disintegration due to medium temperature variation, such as in the gastrointestinal tract. In order to investigate the combined effects of the disintegration medium temperature and compaction pressure on the tablet disintegration time, contour plots were constructed (Figure 4). For the investigated formulations, there was no statistically significant interaction effect found between compaction pressure and the medium temperature (*p* = 1.00). Compaction pressure did not affect disintegrant sensitivity to variations in the medium temperature. Nevertheless, increased compaction pressure did result in increases in the disintegration time, whilst higher median temperature decreased the disintegration time, albeit not as remarkable for tablets with SSG (Figure 4a).

### 3.2. Tablet Disintegration with and without Preheating of Tablets

**Figure 5:**
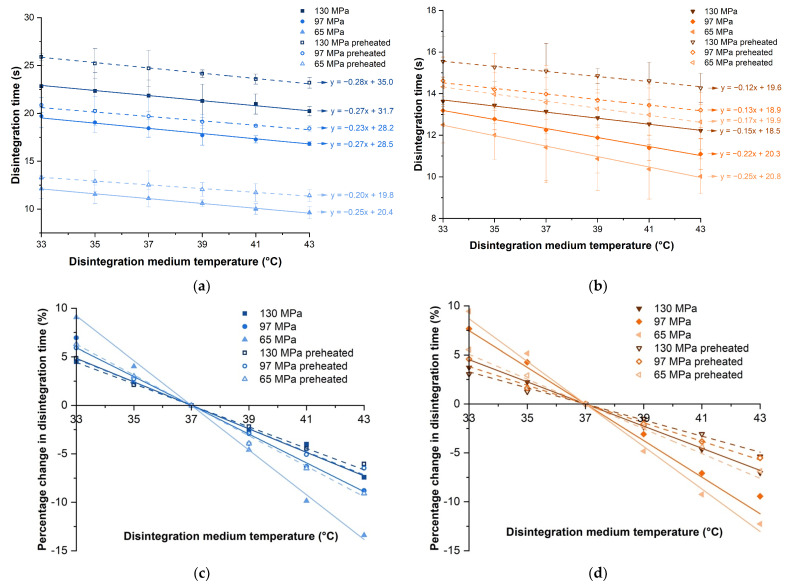
Plots of disintegration time against disintegration medium temperature for tablets containing (**a**) SSG and (**b**) XPVP, with or without preheating, produced at different compaction pressures; plots of percentage change in disintegration time against disintegration medium temperature for tablets consisting of (**c**) SSG and (**d**) XPVP, with and without preheating, produced at different compaction pressures. Note: All trend lines drawn are best fit lines.

For tablets with short disintegration times, the tablets could have partially disintegrated before they attained the temperature of the medium, thereby decreasing the full extent of the differences in disintegration time obtained at different medium temperatures. Thus, tests using preheated tablets at the same temperature as the medium were conducted, in order to ensure the test tablets established a temperature equilibrium with the medium instantaneously, and could provide an unambiguous effect of the medium temperature on disintegration time. For the purpose of comparison, plots of disintegration time against the disintegration medium temperature, with and without preheating of tablets, were constructed and the best fit lines drawn (Figure 5a,b).

Faster tablet disintegration was observed at higher medium temperatures for all tablets, and preheating of tablets resulted in significantly slower tablet disintegration (*p* < 0.001). The gradient of the best fit line was generally gentler for preheated tablets compared to the corresponding tablets that were not preheated, indicating that preheating reduced the temperature sensitivity of disintegrants.

#### 3.2.1. Percentage Change in Disintegration Time Due to Medium Temperature

The percentage change in disintegration time was calculated in comparison to the corresponding tablets disintegrated at 37 °C and plotted (Figure 5c,d). The percentage change in the disintegration time with medium temperature for preheated tablets was also linear. The gradient ranged from −1.6 to −0.8% per degree temperature change, which was lower than that of tablets that were not preheated. Nevertheless, the sensitivity of both SSG and XPVP to variations in the medium temperature is further confirmed to be similar. The effect of medium temperature on tablet disintegration was also less pronounced at higher compaction pressures, regardless of whether the tablets were preheated.

#### 3.2.2. Liquid Penetration Rate into Tablets

**Figure 6:**
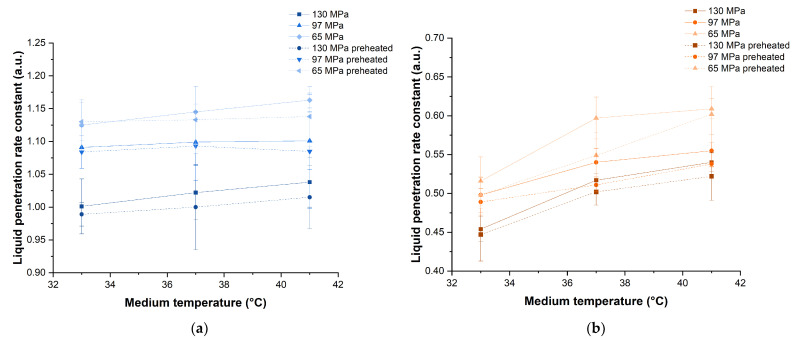
Plots of liquid penetration rate constant against medium temperature for tablets containing (**a**) SSG and (**b**) XPVP, with or without preheating, produced at different compaction pressures.

The liquid penetration rate constant was plotted against the medium temperature for tablets with SSG or XPVP, with or without preheating, produced at different compaction pressures (Figure 6). Liquid penetration appeared slower for preheated tablets compared to unheated tablets, but this was not found to be statistically significant (*p* = 0.405).

As discussed previously for tablets that were not preheated, the liquid penetration rate into preheated tablets increased as the medium temperature increased. The effect of the medium temperature on liquid penetration was significant for preheated tablets with XPVP (*p* = 0.001) but not SSG (*p* = 0.960). Liquid penetration was slower at higher compaction pressures, and this was found to be significant for preheated tablets with SSG (*p* < 0.001), but only borderline significant for those with XPVP (*p* = 0.051). It was noted that the trends observed with tablets with and without preheating were generally similar.

### 3.3. Investigating the Effect of Preheating on Tablets

**Figure 7:**
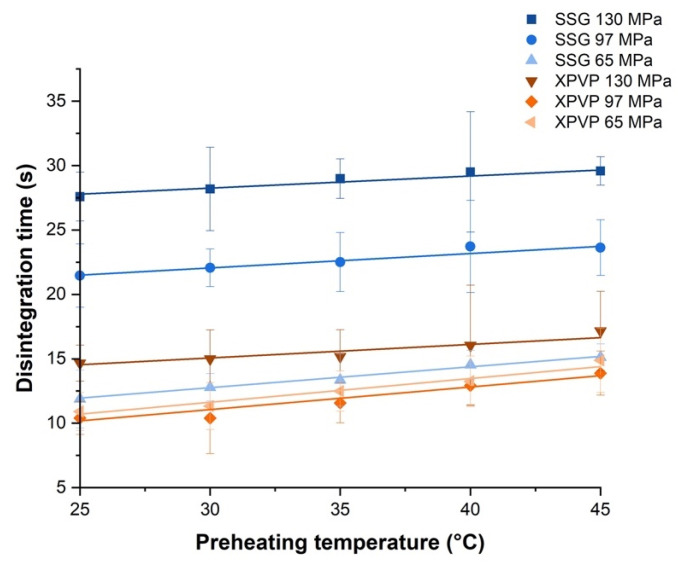
Plots of disintegration time at 37 °C against preheating temperature for tablets made with SSG or XPVP, produced at different compaction pressures. Note: All trend lines drawn are best fit lines.

Interestingly, preheating of tablets resulted in slower tablet disintegration. In order to further understand this phenomenon, tablets were preheated to different temperatures prior to the disintegration test at 37 °C (Figure 7). From the positive slope of the plots (gradients ranging from 0.09 to 0.18), it can be inferred that the tablet disintegration time increased slightly as the preheating temperature increased.

#### 3.3.1. Moisture Content

Preheating of tablets could have altered the moisture content of the tablets, which might then affect their disintegratability. The moisture contents of the tablets, with or without preheating, was determined (Table 2). The tablets were all found to have low moisture content (≤1%), with no significant difference between tablets with and without preheating (*p* = 0.95). Hence, the short preheating time did not cause any substantial moisture loss from the tablets and therefore, moisture content variability could not be a major contributing factor.

#### 3.3.2. Change in Physical State of Disintegrant Particles

A recent study by Bauhuber et al. [38] found that storage at an elevated temperature, without high humidity, triggered a moderate strain recovery of XPVP. The researchers postulated that a plasticization effect by an elevated temperature caused a premature release of stored energy, reducing the disintegrant efficiency of XPVP when it was subsequently exposed to water. Similarly, preheating of tablets in the oven in this study could be responsible for the partial strain recovery of XPVP or affected the physical state of disintegrant particles. This could potentially contribute to a smaller volumetric expansion of the disintegrants when wetted and therefore, prolonged the disintegration process as the preheating temperature of tablets increased.

In order to determine if the plasticization of disintegrant particles (or other mechanisms affecting the physical state of the disintegrant particles) were responsible for the observations in this study, it was of interest to determine if the effect of preheating was reversible. The temperature and duration of the preheating phase was varied, and the tablets subsequently tested for disintegration. Another set of tablets were allowed to cool to room temperature before the disintegration test. The disintegration time of tablets that were not subjected to any heat treatment was determined as well.

**Figure 8:**
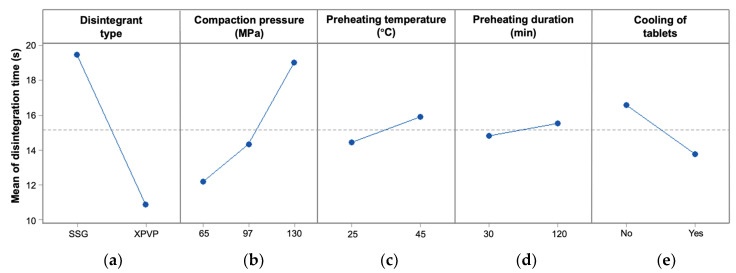

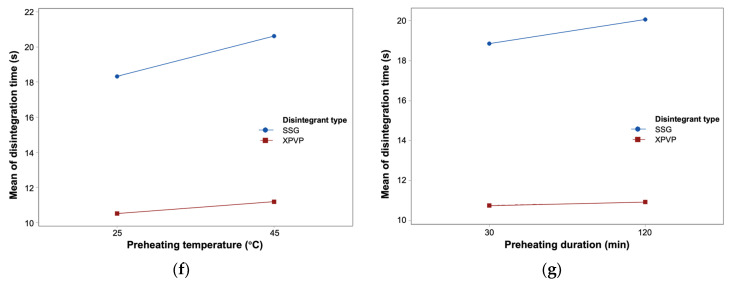
(**a**–**e**) Main effects plots of disintegration time for tablets subjected to different heat treatments; interaction plots of (**f**) preheating temperature and disintegrant type, and (**g**) preheating duration and disintegrant type. Note: The dotted line indicates the average disintegration time of the tablets in this study.

As shown in Table 3, preheated tablets generally showed slower disintegration than unheated tablets. Preheating the tablets at higher temperatures or for longer durations resulted in slower tablet disintegration (Figure 8), indicating that heat might have a detrimental effect on disintegrant functionality. When the preheating temperature increased from 25 to 45 °C, tablet disintegration time increased by about 10% (Figure 8c). The difference in tablet disintegration time was significant for both SSG (*p* = 0.027) and XPVP (*p* = 0.011), with a greater change observed for tablets with SSG (Figure 8f). When the preheating duration was increased from 30 to 120 min, tablet disintegration time increased by about 6.4% for tablets with SSG, but only 1.6% for tablets with XPVP (Figure 8g). The effect of the preheating duration was not found to be statistically significant (*p* = 0.24 for SSG and *p* = 0.51 for XPVP). Hence, tablet disintegratability was more sensitive to changes in the preheating temperature than the duration of preheating.

Cooling of the tablets to room temperature prior to the disintegration test decreased the tablet disintegration time (Figure 8e, *p* < 0.001). In fact, the disintegration time seemed to return to baseline values (i.e., without any heat treatment) for most of the tablets that were cooled prior to the disintegration test (Table 3). The difference in disintegration time between unheated tablets and cooled tablets was not found to be significant (*p* = 0.82 for SSG and *p* = 0.16 for XPVP). Hence, it could be inferred that the effect of preheating on disintegration time was reversible, and that plasticization of the disintegrant particles was unlikely to account for the effect of preheating observed.

#### 3.3.3. Temperature Difference between Tablet and Disintegration Medium

It was likely that the temperature difference between the tablet and the disintegration medium (ΔT) had an effect on tablet disintegratability. It has been postulated that a temperature difference alters the fluid flow due to density change [39]. As the temperature difference between hot and cold areas of fluid increases, the flow eddies developed could contribute to the energetics of liquid flow in the vicinity. Convection occurs when particles with more heat energy in a liquid move and take the place of particles with less heat energy [40]. This is consistent with the results where disintegration of unheated tablets was clearly faster in the disintegration medium of the higher temperature (Figure 5). It is therefore likely that ΔT provided the temperature gradient which translated into flow velocity or liquid penetration rate into the tablets, contributing to faster disintegration and thus, reducing the disintegration time.

### 3.4. Understanding the Effect of Temperature & Future Research Directions

In practice, tablets are not normally preheated prior to consumption. However, the results suggest that tablets stored under colder conditions (e.g., during winter or in a refrigerator) would disintegrate faster than tablets stored in warmer conditions (e.g., during summer or in a heated car). With regards to consuming tablets with warm or cold food and drinks, the tablet disintegration time could be up to 12% faster at 43 °C, and 32% slower at 21 °C. This difference in tablet disintegratability could be significant for tablets with slower disintegration (e.g., those compacted at higher pressures or containing a substantial portion of a hydrophobic drug or excipient) and warrant further studies.

In this study, placebo tablets consisting of a hydrophilic filler were produced, and they did not contain an active pharmaceutical ingredient. In reality, the drug could be hydrophilic and highly sensitive to the process of water conduction. As a result, changes in fluid flow would affect tablet disintegration and drug release. The influence of the disintegration medium temperature could be relevant for drug formulations where fast tablet disintegration and drug onset are pertinent. In such cases, it may be advisable for patients to avoid consuming these medications with cold water. However, for hydrophilic tablet formulations such as those investigated in this study, the disintegrant efficiency was not highly sensitive to temperature variations. Disintegrants acting via swelling or strain recovery also showed similar sensitivity to variations in the disintegration medium temperature. Future studies should investigate the temperature sensitivity of other disintegrants and formulations to improve confidence when selecting disintegrants in situations where there is concern about tablet disintegration profile variability due to temperature. Similarly, the intra-oral temperature has also been found to vary considerably (18.9 to 48.4 °C) with meal temperatures [41], suggesting that the effect of the disintegration medium temperature may be significant for orodispersible tablets as well.

A better understanding of the ΔT effect could also allow for the development of novel strategies to boost disintegrant functionality and improve tablet disintegratability. Future studies can explore modulating the tablet microenvironment temperature to influence tablet disintegratability via the ΔT effect. For example, excipients that react exothermically with water could be incorporated into tablets to increase the tablet microenvironment temperature. This may be of particular value when designing tablets meant for rapid disintegration.

It has also been reported that water activity affects tablet wetting and consequently, tablet disintegration [42]. Water activity refers to the availability of free moisture for reaction, and water flows from regions with higher water activity to regions with lower water activity [43,44]. This could result in a phenomenon similar to the ΔT effect during tablet disintegration. Starch and cellulose-based materials generally have lower water activity, whereas excipients such as DCP and lactose have relatively higher water activity [43,44,45,46]. Future studies should also explore the effect of a difference in tablet microenvironment water activity on tablet disintegratability.

## 4. Conclusions

As superdisintegrants are increasingly used in tablet formulations, it is important to understand how their disintegrant function and tablet disintegratability may be affected by variations in temperature and compaction pressure. This study provided deeper insights into the function of temperature and compaction pressure on the disintegratability of tablets formulated with superdisintegrants. Tablet disintegration was faster in disintegration media maintained at higher temperatures. Despite having different mechanisms of action, the disintegrant functionality of SSG and XPVP was affected by variations in medium temperature to similar extents, with no significant differences in temperature sensitivity. The compaction pressure also did not affect disintegrant sensitivity to variations in medium temperature. Preheating of tablets resulted in slower tablet disintegration, but the effect of heat treatment on tablet disintegration was found to be reversible and unlikely to be attributed to changes in the physical state of the disintegrants. It is therefore likely that the temperature difference between the tablet and the disintegration medium (ΔT) affected the rate of fluid flow into the tablets as eddies developed, hence influencing the tablet disintegration time. Understanding the ΔT effect would be useful during product design when formulation decisions are made, as well as in therapeutics when tablets are consumed.

## Figures and Tables

**Table 1:** Tensile strength and porosity of tablets produced.

Disintegrant	Compaction Pressure (MPa)	Tensile Strength (MPa)	Porosity (%)
SSG	65	0.35 ± 0.01	27.1 ± 0.2
	97	0.57 ± 0.02	23.8 ± 0.3
	130	0.82 ± 0.03	21.6 ± 0.1
XPVP	65	0.24 ± 0.02	28.0 ± 0.3
	97	0.46 ± 0.02	24.2 ± 0.3
	130	0.67 ± 0.02	22.0 ± 0.3

± standard deviation.

**Table 2:** Moisture content of tablets.

Disintegrant	Conditioning	Moisture Content (%)
SSG	Not preheated	0.33 ± 0.07
	Preheated to 25 °C	0.34 ± 0.04
	Preheated to 45 °C	0.36 ± 0.09
XPVP	Not preheated	0.52 ± 0.12
	Preheated to 25 °C	0.52 ± 0.14
	Preheated to 45 °C	0.50 ± 0.08

± standard deviation.

**Table 3:** Disintegration time of tablets containing SSG or XPVP, produced at different compaction pressures and subjected to different heat treatments.

	Disintegration Time (s)					
	SSG			XPVP		
Heat Treatment	65 MPa	97 MPa	130 MPa	65 MPa	97 MPa	130 MPa
None (i.e., unheated)	12.0 ± 1.6	17.9 ± 1.2	22.4 ± 2.3	9.8 ± 1.3	8.9 ± 0.3	11.2 ± 0.0
25 °C, 30 min	12.3 ± 1.2	18.4 ± 4.3	29.2 ± 0.3	10.6 ± 1.0	9.3 ± 0.3	12.3 ± 1.9
25 °C, 30 min, cooled	11.7 ± 2.0	16.7 ± 2.2	20.4 ± 1.0	10.3 ± 1.0	8.7 ± 2.2	11.4 ± 0.1
25 °C, 120 min	15.0 ± 0.4	18.6 ± 2.5	31.2 ± 5.2	10.8 ± 1.3	9.4 ± 0.1	12.5 ± 1.1
25 °C, 120 min, cooled	12.4 ± 1.2	16.7 ± 2.5	20.6 ± 7.3	10.2 ± 1.0	8.7 ± 0.7	12.0 ± 1.0
45 °C, 30 min	14.3 ± 0.9	21.5 ± 0.5	30.0 ± 4.8	11.2 ± 0.5	11.3 ± 0.5	13.5 ± 2.2
45 °C, 30 min, cooled	12.7 ± 2.1	17.9 ± 1.1	21.2 ± 7.1	10.4 ± 0.9	8.7 ± 0.9	11.5 ± 0.2
45 °C, 120 min	16.6 ± 1.3	23.0 ± 1.9	33.0 ± 11.8	11.5 ± 0.2	11.5 ± 0.3	14.1 ± 1.1
45 °C, 120 min, cooled	14.6 ± 1.2	19.6 ± 1.3	23.0 ± 5.8	10.1 ± 1.1	8.8 ± 1.2	11.5 ± 0.2

± standard deviation.

## Data Availability

The data presented in this study are available in the article.
